# On the Use of Laser-Induced Graphene (LIG) in the Development of Chemoresistive Gas Sensors

**DOI:** 10.3390/s26061934

**Published:** 2026-03-19

**Authors:** Alejandro Santos-Betancourt, Xavier Vilanova

**Affiliations:** Universitat Rovira i Virgili, Microsystems Nanotechnologies for Chemical Analysis (MINOS), Departament d’Enginyeria Electronica, Països Catalans, 26, 43007 Tarragona, Spain; alejandro.santos@urv.cat

**Keywords:** laser-induced graphene, LIG, laser parameters, LIG-based gas sensor, LIG combined with MOX/TMD/NP/polymers, LIG in heaters, LIG as IDEs

## Abstract

In recent years, two-dimensional (2D) materials have attracted growing attention for their application in chemoresistive gas sensors. Among these materials, graphene stands out due to its exceptional electrical, mechanical, and chemical properties. A simple and low-cost method for producing graphene involves the use of a laser to induce its formation on carbon-rich substrates, such as polyimides. This technique, first introduced in 2014, has been successfully applied in the fabrication of various types of sensors, including pressure sensors, temperature sensors, biosensors, and gas sensors. For chemoresistive gas sensors, laser-induced graphene (LIG) has been used either as an electrode or as part of the nanocomposite forming the active sensing layer. Moreover, this technology has allowed the use of heating elements. Sensing performance, including sensitivity and selectivity, can be tailored by incorporating different materials into the nanocomposite, such as metallic nanoparticles, metal oxides, or conductive polymers. These modifications can be implemented using low-cost and scalable fabrication methods, making this approach highly suitable for the development of affordable and efficient gas sensors. In this contribution, we present a comprehensive overview of the contributions, reported from the proposal of LIG technology in 2014 to 2025, about the use of this fabrication process in the development of chemoresistive gas sensors.

## 1. Introduction

The development of gas sensors with high sensitivity, rapid response capacity, and low power consumption is very important for applications in areas such as environmental monitoring, industrial safety, and medical diagnostics. Among the many different types of gas sensors, chemoresistive ones have attracted a great deal interest for their simplicity and the simple electronics required for their use. In this context, graphene has emerged as a highly promising material for gas sensing due to its exceptional structural, electronic, and surface properties. As a two-dimensional material composed of a single layer of sp^2^-bonded carbon atoms arranged in a hexagonal lattice, graphene exhibits an extremely high specific surface area, enabling efficient interaction with adsorbed gas molecules. These interactions typically involve charge transfer processes that induce measurable changes in graphene’s electrical conductivity. Also, graphene allows the detection of gas species at very low concentrations, potentially down to the single-molecule level. In addition, graphene’s high carrier mobility, low electrical noise, and ability to operate at room temperature provide significant advantages over conventional metal-oxide-based gas sensors.

Although graphene is widely studied as a theoretical two-dimensional crystal, it was once considered thermodynamically unstable. The experimental demonstration of graphene’s stability and its unique electronic properties in 2004 by Andre K. Geim and Konstantin S. Novoselov [1] marked the beginning of extensive research into two-dimensional materials.

Since graphene’s discovery, significant effort has been devoted to developing reliable and scalable methods for producing it. These methods can be broadly classified into top-down and bottom-up approaches. Top-down techniques, such as mechanical exfoliation and liquid-phase exfoliation of graphite, rely on the separation of graphene layers from bulk graphite. Mechanical exfoliation, the procedure used by A. K. Geim and K. S. Novoselov, produces graphene with outstanding crystalline quality and minimal defect density, making it ideal for fundamental studies. However, it is not suitable for large-scale fabrication. Liquid-phase exfoliation offers improved scalability and enables the production of graphene flakes in large quantities, although the resulting material often exhibits a broad thickness distribution and increased defect density. Another widely used top-down approach involves the chemical oxidation of graphite to produce graphene oxide, followed by exfoliation and subsequent reduction to obtain reduced graphene oxide. This method is attractive due to its low cost and scalability, but the presence of structural defects and residual oxygen-containing functional groups generally limits the electrical performance of the resulting material.

Bottom-up approaches, particularly chemical vapor deposition (CVD) on metal substrates such as copper or nickel, have become crucial to the fabrication of graphene for electronic and sensing applications. CVD enables the growth of large-area, continuous graphene films with controlled thickness and relatively high crystalline quality. Despite the need for high processing temperatures and additional transfer steps, CVD-grown graphene is widely regarded as one of the most suitable forms for gas sensor devices. Alternative bottom-up methods, such as epitaxial growth on silicon carbide via controlled silicon sublimation, allow the direct formation of high-quality graphene on insulating substrates, albeit with higher costs and limitations in sample size.

In 2014, the group directed by J.M. Tour proposed a new procedure for obtaining graphene by laser ablation of a polyimide substrate [2] named laser-induced graphene (LIG). After that, this procedure was extended for other substrates with a high carbon content, such as paper, wood, and polymers [3,4,5]. This procedure allows the obtention of graphene in an easy and economical way, making it very interesting in regard to the series production of chemoresistive gas sensors. In 2019, this same group proposed, for the first time, to use this methodology for the synthesis of a chemoresistive gas sensor [6]. They explored the direct growth of LIG on a polyimide substrate and transferred this layer onto a cement substrate via the peeling-off method. They observed a change in the electrical resistance of the LIG layer when the layer was exposed, starting from a vacuum, to air, helium, or oxygen in a reproducible way. They observed that the change in resistance was related to the CO concentration balanced in He.

The combination of graphene’s sensing capabilities with advances in scalable production techniques has established it as a key material platform for next-generation gas sensors. Further improvements in device performance can be achieved through surface functionalization, doping, and hybridization with metal nanoparticles or other two-dimensional materials, enabling enhanced sensitivity, selectivity, and long-term stability.

## 2. Key Parameters for the Production of LIG

Although a CO_2_ laser operating in the infrared region was used in most of the reported work devoted to the obtention of LIG for gas-sensing purposes, the use of UV lasers has also been reported, such as one study where an LIG layer was used as a VOC sensor for environmental monitoring [7]. While for CO_2_ lasers, the synthesis of graphene is based on a thermochemical process, for UV lasers, it is based on an optochemical process.

Several laser parameters can be adjusted to obtain the desired LIG, and these parameters affect its quality. The main ones are laser power and scan speed, together with the frequency of the laser pulse (in pulsed lasers) and laser spot size. These parameters determine the power density applied to a specific point of the substrate. If the applied power density is too low, the substrate can be altered in some way, but graphitization will not be achieved. Consequently, graphene will not be created. On the other hand, if the applied power density is too high, the substrate will be burned or even destroyed. Accordingly, only a correct combination of laser parameters can lead to the formation of LIG, but depending on the total power density applied, the morphology of the layer and electrical parameters such as sheet resistance can be tuned [8].

**Figure 1:**
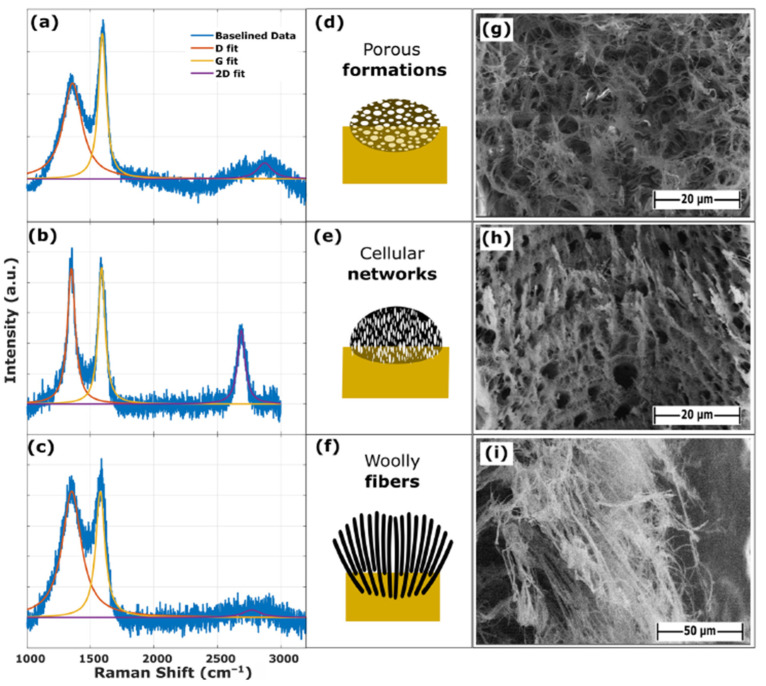
Raman spectra of three different morphologies (**a**–**c**); a schematic of morphologies (**d**–**f**); and FESEM images (**g**–**i**) (from [8]).

Using a CO_2_ laser system (SYNRARD 48-2, Novanta Precision Manufacturing, New York, USA) with a wavelength of 10.6 μm, a maximum power of 25 W, a maximum marking speed of 5000 mm/s, and a frequency range from 0 Hz to 20 kHz, 6.0 × 0.6 mm^2^ strips of LIG were synthesized on a polyimide substrate. The impact of variation in laser parameters on the quality and morphology of the LIG was analyzed using SEM and Raman. The morphologies of the LIG were classified into three different types, namely, types with porous formations, cellular networks, and woolly fibers, as shown in Figure 1.

LIG samples with the cellular network morphology were obtained with low laser power (5–10%) and a low scanning speed (50–200 mm/s), LIG with the woolly-fiber morphology was acquired with medium-high laser power (30–50%—a power level over 50% caused the substrate to burn) and a medium scanning speed (200–600 mm/s), and LIG with porous formations was obtained using medium laser power (20–40%) and a high scanning speed (600–1000 mm/s).

Most samples with the porous formation morphology exhibited sheet resistance greater than 2 kΩ/sq, while the samples with the cellular network morphology had values lower than 2 kΩ/sq. In the case of the samples with a woolly-fiber morphology, their sheet resistance showed a great degree of dispersion, but the majority of samples corresponded to the intermediate resistance range (0.2–2 kΩ/sq).

Another parameter to be considered is the distance between two consecutive lines of ablation. A great distance can lead to a discontinuous LIG layer, while a short distance can cause overburn due to the combined effect of two consecutive lines. This parameter is closely related to beam diameter. The greater the beam diameter, the greater the distance between two consecutive lines. As reported in [9], if the flow of current is perpendicular to the direction of the laser scans, decreasing the distance between consecutive scans will lead to a more conductive path.

Finally, defocusing the laser beam can affect the power density applied to the substrate, leading to wider strips of LIG for each scan, which can lead to a more homogeneous layer.

## 3. LIG in the Development of Chemoresistive Gas Sensors

**Figure 2:**
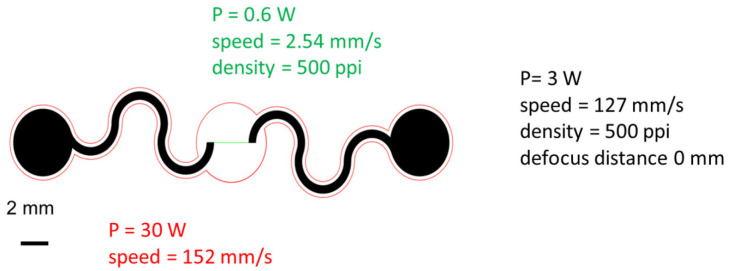
Pattern of the LIG with the corresponding laser parameters.

Graphene, like other carbon-based active layers used for chemoresistors, shows a good response to NOx compounds. Accordingly, LIG can be used directly as a sensing layer for this gas, as can be seen in [10]. An LIG-based gas sensor is designed such that it consists of a straight LIG sensing region that is 150 μm in width; a serpentine Ag/LIG electrode; and a 0.5 mm wide soft elastomeric substrate (500 μm-thick Ecoflex). In this way, the sensing region has much higher resistance, causing localized Joule heating. A 10 μm thick semipermeable PDMS membrane is spin-coated on the top of the sensor to encapsulate it and provide moisture-resistant properties. The process starts by attaching the polyimide film to a glass with water-soluble tape. Laser treatment of the polyimide, with different setting parameters, is then performed, as reflected in Figure 2. Here, green denotes the sensing region, black denotes the electrodes, and red denotes the edges that are cut to free the structure from the substrate when submerged in water. The free-standing structure is deposited onto the Ecoflex substrate, and the electrode regions are covered with Ag ink. Finally, the PDMS membrane is spin-coated on the top of the sensor.

Analyzing the effects of both laser power and image density in the sensing region on sensor response, the authors found that the best combination was the one that exhibited the highest BET area, with a needle-like morphology of the LIG layer. Analysis of the sensor’s behavior confirmed that the LIG behaved like a p-type semiconductor. The best sensor showed a response of 4‰, fast responses/recovery, and recovery times ranging from 113/296 s to 1 ppm NO at room temperature (with a bias voltage of 0.05 V), being selective with respect to NOx (it also produced a response to both NO and NO_2_ and a much weaker response to other gases such as acetone, methanol, ethanol, ammonia, and carbon dioxide at higher concentrations). Comparing the results of sensors with and without the PDMS membrane, it was observed that PDMS incorporation made the sensor insensitive to changes in ambient humidity.

As is well known, the response to NOx or to other gases can be further improved by doping/decorating the LIG. The LIG-doping/decorating process has been carried out using different types of dopants, such as metallic nanoparticles, polymers, metal oxides, transition metal dichalcogenides, or a combination of these dopants. Different methodologies have also been reported for this process. Moreover, LIG has been used for the structural elements of gas sensors, such as the electrodes or heating element. In this review, all the possibilities reported thus far will be discussed.

### 3.1. MOX/TMD-LIG Combinations

Several approaches have combined both transition-metal dichalcogenides and metal oxides, sometimes including metallic NP as well, with LIG. For instance, L. Yang et al. [11] used a structure similar to the one proposed in [10] to obtain a self-heated gas sensor, but in this case, the LIG sensor layer was doped by drop-casting a mixture of reduced graphene oxide (rGO) and molybdenum disulfide (MoS_2_). Applying voltages of up to 12 V, the researchers heated the sensing layer to 80 °C. The optimal temperature was found to be 60 °C, leading to a response (R/Ro) of 6‰ for 1 ppm of NO_2_, good selectivity for NOx, a theoretical limit of detection (LOD) of 1.2 ppb, and response/recovery times of 360/720 s, respectively.

J. Zhao et al. [12] proposed a completely different way of doping LIG. The methodology proposed starts with preparing an LIG layer, in the form of a straight line, over a polyimide substrate. After that, the precursors of the desired doping are drop-casted on the previously ablated strip of LIG, and a second laser treatment (with lower power density than the first one) is used to convert the deposited precursors into the desired doping material. The final doping materials, along with the corresponding precursors used, are listed in Table 1.

MoS_2_ doping led to the best response to NO_2_ (ΔR/Ro = 12.5‰ aprox.). CuO led to the best response for H_2_S (ΔR/Ro = 10‰ aprox.), while Ag/ZnO led to the best response for TMA.

The same approach—namely, deposition via drop-casting aqueous solutions of the precursors of the desired dopants on a previously formed LIG layer strip (0.24 × 8.0 mm), with contact pads (0.3 × 0.3 cm^2^) at each extreme, followed by a second laser treatment to obtain the doping elements from their precursors—was explored by L. Zhang et al. [13]. In this case, the dopants considered were MoS_2_ (with (NH_4_)_6_Mo_7_O_24_·3 H_2_O as a precursor), Au NPs (with HAuCl_4_·3 H_2_O as a precursor), and a combination of both dopants for NO_2_ sensing. Co-decoration with Au and MoS_2_ led to the best response of 11.14‰ for 1 ppm of NO_2_ when the sensor was operated at 90 °C (achieved by self-heating of the LIG layer when a measurement voltage was applied to the connection pads), with an LOD of 3.6 ppb and good selectivity.

A slightly different approach was used to dope a 1.0 × 1.0 mm^2^ LIG area with MoOx using metal–organic decomposition (MOD) [14]. The LIG layer was obtained through the laser irradiation of a polyimide film on soda glass using a YVO_4_ laser marker. To find the optimal laser parameters, fluences ranging from 1.273 to 127.3 J cm^−2^ were tested at a scanning speed of 1000 mm s^−1^ in air. Instead of the use of a second laser treatment, 100 µL of the precursor (ammonium molybdate, citric acid, and N-dimethylformamide) was spin-coated onto the LIG layer and sintered in an electric furnace at 673 K for different amounts of time. A gas-sensing test revealed that the gas sensor could selectively detect various VOCs (methanol, ethanol, 1-propanol, and 2-propanol) and stably detect low concentrations of methanol at 44.7 ppm when it was operated at 300 °C

A completely different approach was proposed by G. Soydan et al. [15]. They covered the surface of a polyimide substrate with SnO_2_ via drop-casting. After that, they proceeded with CO_2_ laser treatment, using a power of 6.88 W, a scanning speed of 40 mm/s, and an image density of 1000 ppi and defocusing the laser beam by 1 mm. The pattern designed was a set of four meanders forming a Wheatstone bridge, where only one of the arms was exposed to the gases, while the others were used to compensate for temperature changes. The inclusion of this SnO_2_ doping increased the sensor’s response to NO_2_ when operated at 50 °C, behaving like an n-type semiconductor (electrical resistance increased when it was exposed to NO_2_).

A similar approach was followed in [16], namely, depositing a Ce oxide precursor (Ce(NO_3_)_3_·6 H_2_O) dissolved in NMP and mixed with liquid polyimide. The resulting paste was spin-coated onto a glass substrate covered with Kapton tape and finally baked at 100 °C. The process was repeated five times to reach the desired thickness, and a final curing step was performed at 200 °C. The resulting layer was treated with a laser to create 1 mm long filaments that served as sensing layers, with connecting pads at each extreme, which were covered with silver ink. Several sensors were produced with different laser parameters, resulting in an electronic nose that was able to discriminate between nine different odorants.

Another approach was used by A. A. Baker et al. [17]: they used an infrared femtosecond laser operated at a wavelength of 1030 nm, a pulse duration of 250 femtoseconds, and a repetition rate of 50 kHz to create a 20 × 20 mm^2^ LIG area on the surface of a polyimide substrate with a laser power of 5.0 W, with the laser focused onto a 100 μm spot size in a crosshatch configuration, employing a Maestro galvanometric scan head (FARO). The scan speed, line spacing, and scanning duration were 40 mm/s, 200 μm, and 2 min, respectively. The texturing process was performed at 0.6 J/cm^2^ fluence. While the first scanning beam heated the PI surface, the second one created a porous LIG surface. Increasing the number of scans resulted in the evaporation of the previously formed porous LIG. The doped sensor showed better responses to ethanol and acetone than the bare LIG, with the Ag doping being the one that led to the best results.

### 3.2. Metallic Nanoparticle (NP) Doping

Besides the approaches previously reported in [13,17] for Au and Ag NPs, respectively, metallic NPs have also been included in LIG layers via the direct drop-casting of an NP suspension and through the evaporation of the corresponding metal. Both palladium [9] and platinum [18] NPs have been reported to be useful for achieving a sensitive and selective hydrogen sensor working at room temperature. In both cases, the LIG pattern is a strip where the NPs were deposited by e-beam evaporation for Pd and drop-casting of an NP suspension in acetone in the case of Pt. However, it is worth noting that evaporation-based deposition may lead to scalability limitations and significantly increase the overall fabrication cost.

In the case of palladium doping, two different substrates were tested. In the first approach, LIG was directly created on a polyimide substrate and then doped with NPs. In the other case, LIG was transferred onto a PET substrate covered by SU-8; after that, the LIG was doped. This second approach led to worse results than the first one.

In the case of Pt doping, the authors explored the effect of increasing the laser power (from 2.8 to 8.0 W—higher power causes substrate combustion), keeping the scanning speed fixed at 100 mm/s and maintaining an image density of 750 ppi for the obtention of LIG on a polyimide substrate. The quality of the LIG in terms of crystallinity increased with the increase in laser power. Once the LIG pattern was ready, 25 µL of a solution of Pt NPs in acetone was dropped onto it and then dried at 50 °C for 5 min. The resulting NPs had diameters of around 3 nm. The sensor produced in this way showed a greater response than the one reported for palladium and very good selectivity.

D. Kwak et al. [19] also used evaporation for doping LIG with metallic NPs. In this case, the LIG was in the form of a meander instead of a straight line. It was achieved with a CO_2_ laser with a power of 4.0 W, a scanning speed of 127 mm/s, and an image density of 500 ppi on a polyimide substrate. The metallic NPs (Ag, Al, Au, Cu, In, and Pd) were deposited by evaporation through a stencil mask on previously obtained LIG. While the pristine LIG layer produced the best response for NO_2_ detection, doping with silver led to the best response for ammonia detection. In both cases, the sensors were operated at 100 °C (heated by means of a ceramic heater) in a N_2_ ambient atmosphere, and the tested concentration was 1000 ppm. In this case, as commented before, the use of an evaporator and a mask can be a bottleneck in large-scale production.

L. Yang et al. [20] also used Ag NPs drop-casted onto an LIG layer in the form of a strip (5.0 × 0.3 mm) for NO_2_ detection. In this case, the substrate was replaced with polyethylene (PET) covered by a solution containing Pluronic F127 copolymer and phenol−formaldehyde resins in ethanol via spin-coating in two steps (850 rpm for 15 s and 1500 rpm for 45 s), and it was cured in a vacuum oven at 150 °C for 2 h. The fabricated Ag/LIG gas sensor achieved a response of −12‰, a fast response/recovery of 40/291 s, and a low detection limit of a few ppb at room temperature. In this case, the procedure was applied to different fabrics (substituting for the PET substrate), resulting in breathable-gas sensors and intelligent clothing, allowing the permeation of air and moisture.

### 3.3. Polymer Doping

Two different polymers, polypyrrole [21] and PANI [22], were electropolymerized in situ, using an LIG layer on a polyimide substrate as an electrode. To obtain a rectangular LIG layer, a CO_2_ laser (λ = 10.6 µm) with 3.0 W of power, a scan rate of 100 mm/s, and a pulse frequency of 12 kHz was used. In both cases, the resultant sensor with an active layer combining LIG and the corresponding grown polymer was quite sensitive and selective to ammonia (working at room temperature). Nevertheless, in both cases, humidity affected the sensor’s response to ammonia. The best response (ΔR/Ro) to 100 ppm of NH_3_ was obtained for the PANI-coated LIG (around 6.5%).

### 3.4. Interdigitated Electrodes (IDEs) and Heating Elements with LIG

Occasionally, the LIG layer is not directly part of the sensing layer. In these cases, the sensor includes a pair of interdigitated electrodes implemented in LIG over which the active layer is deposited. In these cases, the LIG electrodes, besides acting as a transducer collecting the charge carriers, also play a role in the sensing mechanism. This was the case in the work reported by L. Yang et al. [23]. The authors sprayed a PANI solution over a pair of interdigitated LIG electrodes. The sensor, operated at room temperature, showed a very good response to ammonia, more than ten times higher than that reported for the electrodeposited PANI [22], with good selectivity.

A similar approach applied using a molybdenum disulfide layer was reported by W. Yan et al. [24]. Different concentrations of MoS_2_ solutions were drop-casted over interdigitated LIG electrodes to form the active layer. The resulting MoS_2_ layer showed a flowerlike nanosphere morphology of the molybdenum disulfide crystals. In all the cases, the sensors were highly selective to NO_2_, reaching, in the best case, a response (ΔR/Ro) of over 70%. A similar approach was presented by Z. Peng et al. [25], who used a CO_2_ laser (λ = 10.6 µm) with a power of 4.8 W and a scanning speed of 300 mm/s to define the pair of LIG interdigitated electrodes over which suspensions with different concentrations of MoS_2_ were sprayed as the active layer. In this case, an LIG area was ablated on the back side of the polyimide substrate. This LIG area was used as a heating element as well as an acoustic alarm when AC voltage was applied. Applying a DC voltage of up to 10 V to the backside LIG, the researchers heated the active layer to 150 °C, and it showed quite good homogeneity.

Although the sensor responses at room temperature reached values of around 90% for 3 ppm of NO_2_ for all the MoS_2_ concentrations tested, the response times were too slow. Also, the sensor behavior in the range from 2 to 5 ppm of NO_2_ was clearly non-linear. Additionally, the resolution was poor. In contrast, when the sensing layer was heated to 150 °C, the responses of the sensors were lower, both the response and recovery times were drastically reduced, and the resolution improved, allowing measuring in the range of hundreds of ppb, and an LOD of 0.317 ppb was derived from the calibration curve.

**Figure 3:**
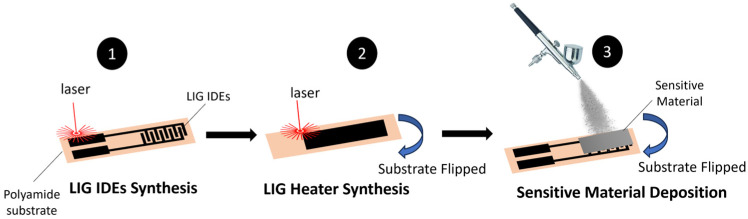
Schematic description of a piece of double-sided laser-induced graphene used to create interdigitated electrodes and a heater.

Figure 3 summarizes the fabrication of a double-sided piece of laser-induced graphene, with the following steps: step 1—LIG-based synthesis of interdigitated electrodes; step 2—LIG-based synthesis of a heater; and step 3—deposition of the sensitive layer.

Another approach to NO_2_ sensing is the use of ZnO nanorods drop-casted onto LIG IDEs [26]. In this case, the authors explored the effect of the ambient conditions when producing an LIG using a fiber laser with a wavelength of 1064 nm. Pulse repetition rate, scan speed, and scan spacing were fixed at 750 kHz, 150 mm/s, and 130 μm, respectively. Accordingly, the polyimide substrate was placed inside a chamber with a controlled atmosphere (vacuum, air, or nitrogen), with a window employed to allow the laser beam to reach the substrate surface. The power density (fluence) applied was varied from 0.376 to 0.567 J/cm^2^ to find the best option. Raman analysis of the resulting LIG in both vacuum and nitrogen atmospheres showed that the ratio of the G to D bands (IG/ID) was higher than that in the case of air, indicating a piece of graphene with fewer defects, and, in fact, the more defective layers showed a better response when exposed to NO_2_, reaching values of 250% at 1 ppm. Although the sensor was quite selective, its response was dramatically affected by the ambient moisture.

An equivalent structure to the one proposed by Peng et al. [25] was used by J. Yang et al. [27] to detect the ethylene emitted by chrysanthemums once harvested using a spin-coated SnO_2_ layer on LIG IDEs. The laser parameters fixed in this case were a power of 9.5 W, a scanning speed of 20 mm/s, and a defusal length of 11 mm. In this case, a linear behavior of the temperature reached with the DC power supplied to the heating element was observed (T = 53.5006·V − 42.5593; r^2^ = 0.9997), reaching temperatures above 300 °C with just 7 V. Regarding the behavior of the sensor, the maximum response (ΔR/Ro = 70% @ 10 ppm ethylene) was obtained for an operating temperature of 250 °C. The calibration curve could be considered linear in three different ranges—0.05 to 1 ppm, 2 to 10 ppm, and 20 to 100 ppm—with an LOD of 1.65 ppb, with the response being unaffected by moisture fluctuations.

For sensing other compounds, different MOX-based active layers have been explored. In [28], the paste prepared was a mixture of sodium dodecyl sulfonate (SDS) with WO_3_, which resulted in a H_2_S sensor with good responses and selectivity. In [29], the layer deposited was sprayed, and the solution was a mixture of SDS with In_2_O_3_ and ZnO. In this case, the active layer was covered by a PDMS membrane to protect it. The resultant sensor showed good responses and selectivity to CH_4_. Finally, in [30], a pair of interdigitated LIG electrodes were covered with a ZnO layer to detect acetone. In this case, the authors used an SBA-15 membrane deposited over the active layer as a moisture barrier film. As a result, the sensor’s response was not affected by humidity, reaching a response of around 25%.

A completely different approach consists of using a nanocomposite as an active layer on top of LIG IDEs. In the case in question, the nanocomposite was composed of multiwall carbon nanotubes (MWCNTs) and cobalt phthalocyanine [31]. A dispersion of this nanocomposite was drop-casted onto LIG IDEs patterned with a CO_2_ laser with a power of 6.0 W, a scan rate of 100 mm/s, and a resolution of 1200 ppi. The sensor, tested for a methanol concentration range from 10 to 1000 ppm, showed a response of 27‰ at the maximum concentration. The sensor was selective to the gas and estimated an LOD of 165 ppb, with response and recovery times of 5 and 108 s, respectively.

Finally, a metallic organic framework (MOF) layer was sprayed over LIG IDEs engraved on a polyimide substrate to form an active layer for NO_2_ detection [32]. A ZIF8@ZnO MOF previously prepared via mechanochemical protocols was dispersed in ethanol so that it could be sprayed. The resulting sensor achieved a response of 78%, with response and recovery times of 58 and 40 s, respectively, for 100 ppm of NO_2_ and showed a lifetime stability of 40 days.

### 3.5. LIG on Non-Flexible Substrates

Although LIG is usually employed to develop sensors on flexible substrates, some researchers have used this approach to develop gas sensors on rigid substrates. In the first case reported, a standard silicon wafer was used as a substrate [33]. Once oxidized, interdigitated electrodes, a temperature-sensing element, and a heater were patterned on the surface using a 30 nm titanium adhesion layer and a 300 nm platinum layer. After an oxygen plasma treatment was applied to the surface, a 10 µm SU-8 layer was deposited and patterned onto the IDEs. This polymer was treated using a CO_2_ laser in air. Ten sequential passes with increasing laser power (10.4, 10.6, and 10.8 W) led to the formation of a reduced graphene oxide. After that, the layer was doped using two different approaches: using SnO_2_ deposited by atomic layer deposition (ALD) and with palladium via electron beam evaporation. The process was finalized with annealing at 350 °C for 12 h.

An rGO sensor was tested under UV light (365 nm) irradiation to measure NO_2_. Increasing light intensity resulted in a lower baseline resistance and sensor response, while the response and recovery time were reduced. A value of 150 mW/cm^2^ was found to be optimal for achieving a sufficient response and reducing the response time. The response was sensitive to humidity changes, reaching a maximum value of 20% at 30% relative humidity for 1 ppm of NO_2_. Doping with SnO_2_ resulted in improved performance in NO_2_ detection when operated under the same conditions. When the sensor was heated, the optimal temperature was found to be 150 °C, reaching a response to 100 ppb of NO_2_ that was above 65%. The response in this case corresponded to an n-type semiconductor, reaching an LOD of 26.2 ppt. When doped with Pd, the sensor was selective to hydrogen, reaching a maximum response of 50% for 10 ppm of H_2_ when operated at 300 °C.

A standard printed circuit board (PCB) with 0.25 mm gap interdigitated electrodes was used as substrate by N. Khattak et al. [34]. They analyzed the difference in performance between a graphene sensor obtained by laser treatment and a graphene layer grown by CVD in the detection of VOCs, such as ethanol, isopropanol, acetone, and ammonia. A piece of CVD-grown graphene on Cu foil in a cold-wall reactor with CH_4_ flow was deposited onto the electrodes in the PCB substrate, while for the LIG, Kraft lignin with water paste was deposited onto and dried on the IDEs and later treated with a 40 W CO_2_ laser engraver with a 1000 dpi resolution at a speed of 100 mm/s. Characterization of the layers indicated that the LIG layer showed more defects than the CVD-grown layer, showing a porous structure, thus allowing a higher surface area, making it more suitable for gas-sensing applications, as confirmed through gas tests. Nevertheless, the response times were quite high.

## 4. Sensing Mechanism for LIG-Based Gas Sensors

After reviewing the variety of use cases of LIG, including different types of hybrid sensing layers, in this section, we aim to provide a coherent physical and chemical description of the gas-sensing mechanism in LIG-based gas sensors.

The electrical response of LIG is typically mediated by surface reactions and interfacial electronic effects, not purely charge transport in the induced graphene. LIG provides a conductive, porous, and mechanically suitable path, but the dominant sensing mechanism is determined by the nature of the functional layer and the interfaces formed with LIG. In ambient conditions, pristine LIG exhibits a p-type behavior, originating from residual oxygen-containing functional groups, structural defects, and adsorbed oxygen species. In chemoresistive operations, exposure to oxidizing gases increases the concentration of holes in the LIG, thereby increasing conductivity. Conversely, reducing gases withdraw holes or react with surface oxygen species, partially reducing holes and altering the carrier concentration of the sensing layer [35].

In hybrid systems, on the other hand, this direct charge-transfer picture is insufficient to fully describe the responses of the sensors when they are exposed to gases. When LIG is combined with n-type metal oxides (MOXs) such as ZnO, SnO_2_, WO_3_, or In_2_O_3_, a p–n heterojunction is formed when the LIG interfaces with the MOX. In ambient conditions, oxygen adsorption on the MOX surface leads to the formation of ionized species (O_2_^−^ and O^−^), extracting electrons from the conduction band and generating a depletion layer. This results in upward band bending in the MOX side and an increased interfacial barrier for charge transport. Exposure to oxidizing gases further depletes electrons, increasing band bending and the height of the barrier. In contrast, reducing gases consume the oxygen species through surface redox reactions, releasing electrons back into the MOX side. This effect narrows the depletion region and lowers the junction barrier. The sensor’s response is therefore dominated by the modulation of the width of the space-charge region of the p-n heterojunction [14,16,36].

Another mechanism applies when LIG is functionalized with transition-metal dichalcogenides (TMDs) such as MoS_2_ or WS_2_. In these cases, gas adsorption occurs preferentially at defect sites, edges, and sulfur vacancies, leading to charge transfer between the gas molecules and the semiconducting TMD. As a result, the Fermi level is shifted and provokes a variation in the resistance in the contact interface between the LIG and TMD. Consequently, the sensing signal appears mainly as a product of the interface-controlled transport rather than because of adsorption on the surface of the induced graphene [13,37].

In NP-decorated LIG (with the NPs consisting of elements such as Au, Ag, Pd, and Pt), the sensing mechanism involves both catalytic surface reactions and electronic barrier modulation. NPs facilitate dissociation or activation of gas molecules and promote the spillover of reactive species toward the underlying sensing layer. At the same time, gas adsorption changes the metal’s work function, modifying the height of Schottky barriers in the interface contacts of the NPs with LIG. These combined effects enhance sensitivity and accelerate the response of the sensor [18,38].

In the case of LIG functionalized with polymers, such as PANI, the sensing mechanism is controlled by chemical doping–dedoping reactions on the polymer side. In conductive polymers, doping–dedoping reactions correspond to reversible protonation–deprotonation processes that modulate the density of charge carriers. This effect leads to significant conductivity changes in the sensitive layer when the sensor is exposed to the gas. Reducing gases induce deprotonation, decreasing polymer conductivity, while LIG provides an electrically percolative pathway and stabilizes the composite structure. For oxidizing gases, the mechanism is the other way around: the conductivity of the polymer is increased because of the protonation effect. The final measured response reflects changes in polymer conductivity coupled with variations in interfacial and contact resistances between LIG and the polymer [22,39]. Table 2 summarizes the sensing mechanism of the LIG combinations during the fabrication of an LIG-based gas sensor.

## 5. Analysis of LIG’s Limitations

Despite the significant progress achieved in the past few years related to the use of LIG to fabricate gas sensors, several challenges still limit its worldwide deployment in practical gas-sensing applications.

First, reproducibility remains a major concern. The structural formation of the induced graphene strongly depends on laser parameters, including those mentioned in this review. But it also depends on the atmospheric conditions during the graphene induction process. Small variations in these parameters can lead to significant differences in porosity, defect density, and the degree of graphitization. These variations might affect the main characteristics of the final sensor; therefore, they must be carefully controlled [2,8].

The second important limitation is the reduced selectivity of pristine LIG [6,7]. As with other graphene-based sensors, their responses are not highly specific to a particular gas species. Therefore, pristine LIG sensors exhibit cross-sensitivity to multiple gases. To overcome this issue, as mentioned in this review, most reported studies relied on functionalization and hybridization strategies [18,21,24,28]. Although these approaches improve sensitivity and selectivity, they also introduce additional steps and complexity in the fabrication process, which might lead the reader back to the first limitation, reproducibility.

The third limitation pertains to the response’s long-term stability, drift, and variability due to changes in environmental parameters such as humidity. Even though some authors claim to have created a sensor that is stable over a long term under laboratory conditions, these three parameters remain open challenges. The highly porous structure of LIG, which is beneficial for gas adsorption, also makes it susceptible to environmental contamination and gradual surface modification during long-term operation. Adsorption of atmospheric species or gradual degradation of functional layers may lead to baseline drift and response degradation over time [25].

All these limitations of LIG are not different from those that hinder other common graphene synthesis techniques. As mentioned above, graphene-based sensors, for example, graphene obtained via CVD, have low baseline sensitivity (unless functionalized with catalytic or selective materials) because of their continuous, low-defect, two-dimensional lattice with fewer active sites. CVD graphene exhibits high carrier mobility and low defect density, producing low noise, good reproducibility, and strong long-term stability when compared with LIG. Its response to gas species is generally faster than that of LIG, and so is its recovery time. But the aspect in which LIG surpasses CVD graphene is its manufacturing process, as it is easier, cheaper, more scalable, and faster and requires less energy and manufacturing equipment.

Finally, the fourth limitation is related to thermal and power management. This must be addressed when LIG is integrated with heating elements for sensor regeneration or temperature-activated sensing layers [11]. Heating the active layer can enhance sensing performance by increasing the response magnitude and reducing both response and recovery times. On the one hand, self-heating strategies offer clear practical advantages. They are easier to implement than integrating a backside heater, which requires precise alignment with the active layer, and, typically, less power is required to reach the desired operating temperature. On the other hand, self-heating inherently limits the effective area of the active region, which may constrain overall sensor response. It is also worth noting that in both scenarios, achieving a uniform and controllable temperature distribution across flexible substrates is not trivial, particularly in miniaturized or wearable devices.

## 6. Conclusions

As shown in the reviewed research, the use of a laser to induce the formation of a graphene layer on top of a substrate (usually a flexible one) is a low-cost technique that has proven to be suitable for the development of the next generation of chemoresistive gas sensors. The graphene layer obtained through this process can be used either as a base for a doped sensing layer or as electrodes, typically interdigitated, with a dual function: acting as a transducer and actively participating in the detection process.

When heating the sensing layer is required for proper operation, possible solutions include self-heating of the laser-induced graphene (LIG) layer or the implementation of a dedicated heating element (fabricated using the same approach) on the backside of the substrate. In all cases, the selection of appropriate laser parameters is crucial to achieving optimal performance for both functions assigned to the LIG.

Also, several approaches have been reported to be able to modify the LIG layer and tune the sensor’s affinity toward the target gas. The results obtained are considered acceptable for many applications. In this context, although certain functionalization routes appear to offer favorable combinations of robustness, thermal stability, and well-defined surface mechanisms, we believe the optimal strategy depends on the target gas, the adsorption pathway, and the operating environment, especially when different materials are combined, such as MOX- or TMD-based systems. From our perspective, efforts should be directed not only towards investigating LIG as an isolated sensing material but also towards studying its interaction with other materials, such as the ones described in this review and others.

Finally, due to the compatibility of LIG with simple fabrication processes, it is a promising method for the next generation of portable, scalable, and application-specific gas-sensing technologies. Although the door is still open for challenges related to reproducibility, selectivity, long-term stability, and thermal management, LIG has shown numerous advantages, such as an extremely low fabrication cost, a simple laser induction process, minimal equipment requirements, and the possibility of producing large-area sensing elements in a matter of seconds.

These benefits substantially lower the entry barrier for mass manufacturing and enable rapid prototyping that is unmatched by more complex synthesis routes such as CVD. Therefore, despite the existing limitations, we believe that with targeted research efforts focused on fabrication process control, surface engineering, and device integration, LIG has the potential to become one of the leading technologies for the mass production of low-cost, next-generation gas sensors.

## Figures and Tables

**Table 1:** Dopants and the corresponding precursors used [12].

Dopant	Precursor
MoS_2_	0.07 M ammonium paramolybdate and 3 M thiourea
CuO	0.5 M copper nitrate
Ag/ZnO	0.03 M silver nitrate and 0.5 M zinc nitrate
In_2_O_3_/Cr_2_O_3_	0.5 M indium nitrate and 0.5 M chromium nitrate

**Table 2:** Summary of dominant sensing mechanisms of LIG-based gas sensors.

Functionalization on LIG	Dominant Electronic Structure	Key Surface Reactions	Dominant Sensing Mechanism
Pristine LIG	p-type behavior	Physisorption/ weak chemisorption	Direct charge transfer
LIG + n-type MOX	p–n heterojunction	Oxygen ionosorption (O_2_^−^, O^−^)	Depletion-layer/ barrier modulation
LIG + TMDs	Semiconductor–conductor junction	Adsorption at defects, edges, and vacancies	Interface-controlled charge transport
LIG + metal NP	Schottky contacts	Catalysis, spillover, and work-function shifts	Barrier modulation/ catalytic amplification
LIG + conductive polymers	Percolative composite	Reversible protonation/ deprotonation	Chemical doping–dedoping of the polymer phase

## Data Availability

Not applicable.

## Abbreviations

The following abbreviations are used in this manuscript:

LIG	Laser-induced graphene
TMDs	Transition-metal dichalcogenides
MOX	Metal oxide
NPs	Metallic nanoparticles
IDEs	Interdigitated electrodes
